# Modified Treatment Approach Using Cardiovascular Disease Risk Calculator for Primary Prevention

**DOI:** 10.1371/journal.pone.0104478

**Published:** 2014-08-13

**Authors:** Himanshu Gupta, Chun G. Schiros, Thomas S. Denney

**Affiliations:** 1 Department of Medicine, Cardiovascular Disease, University of Alabama at Birmingham, Birmingham, Alabama, United States of America; 2 VA Medical Center, Birmingham, Alabama, United States of America; 3 Department of Electrical and Computer Engineering, Auburn University, Auburn, Alabama, United States of America; University of Bologna, Italy

## Abstract

**Background:**

The recent guidelines for preventing atherosclerotic cardiovascular events are an important advancement. For primary prevention, statins are recommended if the ten-year risk is ≥ 5% (consideration for therapy) or ≥ 7.5% (definitive treatment unless contraindication after discussion). We rationalized that a significant cohort with ten-year risk below the treatment thresholds would predictably surpass them within the recommended 4–6 year window for reassessing the ten-year risk. As atherosclerosis is a progressive disease, these individuals may therefore benefit with more aggressive therapies even at baseline.

**Methods and Findings:**

We used publicly available NHANES dataset for ten-year risk calculation. There were 1805 participants. To evaluate the ten-year risk change at five years, we considered two scenarios: no change in the baseline parameters except increased age by five (No Change) and alternatively 10% improvement in systolic BP, total and HDL-c, no smoking with five-year increase in age (Reduced Risk Profile). Amongst non-diabetics with <5% risk at baseline, 35% reached or exceeded 5% risk in five years (5% reached or exceed the 7.5% risk) with No Change and 9% reached or exceeded 5% risk in five years (none reached 7.5% risk) with Reduced Risk Profile; furthermore, 94% of the non-diabetic cohort with baseline risk between 3.5%–5% would exceed the 5% and/or 7.5% boundary limit with No Change. Amongst non-diabetics with 5–7.5% baseline risks, 87% reached or exceeded 7.5% with No Change while 30% reached or exceeded 7.5% risk with Reduced Risk Profile.

**Conclusions:**

A significant population cohort at levels below the treatment thresholds will predictably exceed these limits with time with or without improvement in modifiable risk factors and may benefit with more aggressive therapy at baseline. We provide an improved risk calculator that allows for integrating expected risk modification into discussion with an individual. This needs to be prospectively tested in clinical trials.

## Introduction

The recent ACC/AHA guidelines for the treatment of blood cholesterol to reduce atherosclerotic cardiovascular disease (ASCVD) risk in adults is an important advancement in the prevention of atherosclerotic cardiovascular disease (ASCVD)[1]. These guidelines have proposed the use of the new-pooled cohort equations to define the individuals likely to benefit from either the initiation of statin therapy in non-diabetics or defining the intensity of statin therapy in diabetics for the primary prevention [1], [2]. For non-diabetics between 40–75 years of age and LDL-c between 70–189 mg/dl who have no clinical ASCVD, if the ten-year risk is ≥7.5%, these individuals should be treated with moderate- to high-intensity statin therapy; and those with ten year risk between 5% to <7.5%, it is reasonable to offer treatment with a moderate intensity statin. For diabetics between 40–75 years of age, all of them should be considered for statin therapy, and those with ≥7.5% ten-year risk, the guidelines recommend that high-intensity statin therapy is reasonable if there is no contraindication. The new guidelines also recommend recalculation of estimated ten-year ASCVD risk every four to six years in individuals aged 40–75 years without clinical ASCVD or diabetes. An important aspect of the new guidelines is the strong focus on discussion amongst the physician and an individual for optimal clinical management including statin therapy. A simple risk calculator based on the new pooled cohort equations is available for download[3].

We rationalized that a significant cohort at borderline ten-year risk that are below the recommended thresholds for statin treatment would predictably surpass the treatment thresholds within the recommended four to six year window for reassessing the ten-year risk. Since it is well known that the pathogenesis of atherosclerosis is initiated at a relatively young age, these individuals may therefore benefit with consideration for more aggressive therapies even at baseline. Here we evaluate the impact of reasonable changes in modifiable risk factors on the predicted ten-year risk with time and also provide a tool for easy application.

## Methods

### Study Dataset

We used publicly available NHANES dataset (2005–2010, Data S1, Data S2)[4]. Participants (n = 1805) with all the variable values required for ten- year risk calculation between ages 40–75 years were included. We also analyzed population cohort by including Hispanic participants (categorized as White for purpose of analysis, total n = 2355, Table S1 and S2). Age is reported based on last birthday (i.e., age in completed years) calculated by subtracting the date of birth from the reference date, with the reference date being the date of contact with an individual. Gender and treatment for hypertension is self reported. Diabetes includes self reported or fasting plasma glucose of ≥126 mg/dL or a hemoglobin A1c ≥ 6.5%. Current smokers are persons who smoked 100 cigarettes and who now smoke every day or some days. Race is self reported based on 1997 Revisions to the Standards for the Classification of Federal Data on Race and Ethnicity[5].Total-c and HDL-c measurements are using standard methods as described[6]. Individuals with self reported coronary artery disease, heart attack (or myocardial infarction), angina and stroke were excluded.

The new pooled cohort equations were implemented in a custom software package (MATLAB, Natick, MA). Predicted ten-year risk for a given set of parameters for the NHANES database (called ‘base’ risk in this paper) was calculated. Simultaneously, ten-year risk after five years was computed under two scenarios: 1) assuming no change in the other parameters except age increase by five years (No Change scenario) and 2) a 10% reduction in total-c and systolic BP, a 10% increase in HDL-c, and no smoking (for those who were smokers, Reduced Risk Profile scenario). We then evaluated changes in the risk classification for participant cohort who were < (less than) 5% and between 5-7.5% at baseline, respectively, to ≥ (greater or equal to) the boundary limits in 5 years with reduced risk profile or alternatively with no change scenario. This analysis was performed to evaluate the portion of people at levels below the treatment threshold of 5% and 7.5% that will predictably exceed these limits with time with or without life style modification. The non diabetic patient cohort with baseline risk <5% were further divided into three portions with ten-year risk <3%, between 3–3.5% and 3.5–5% and similar analysis were performed to the ten-year risk between 3–3.5% and 3.5–5% patient cohort. The ten-year risk between 3%–3.5% defines a region where there is no net benefit of moderate statin therapy based on the analysis described in the guidelines[1].Comparisons between the reduced risk profile scenario vs. no change scenario were performed using Fisher's Exact Test (SAS 9.4). A P<0.05 was considered statistically significant. Our version of risk calculator and instruction is available online (Calculator S1, Calculator Instruction S1).

## Results

**Figure 1:**
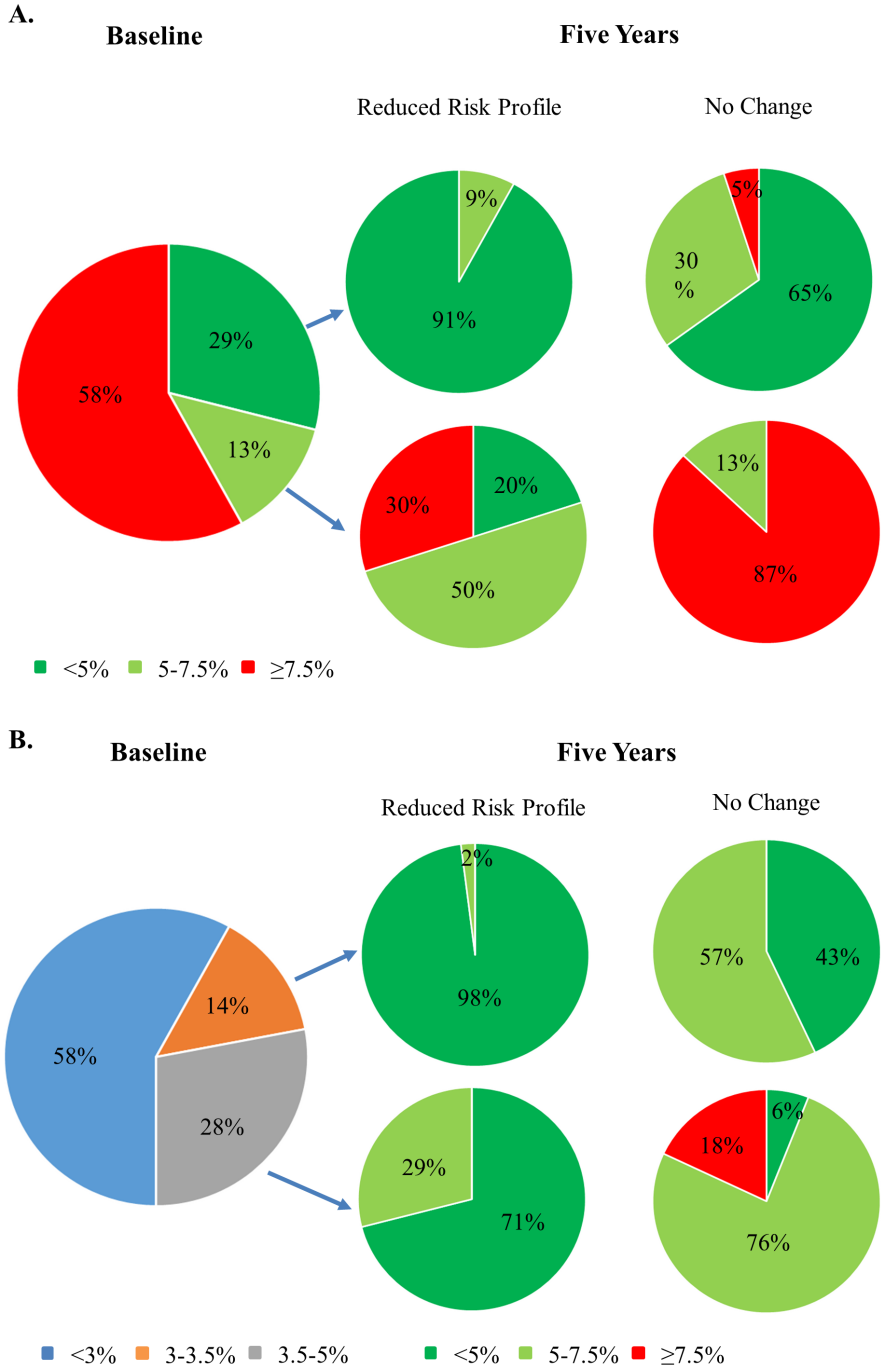
Pie chart demonstrating the impact of risk modification on predicted ten-year risk based on non-diabetic cohort. **A**. At baseline, 29% of the non-diabetic cohort have predicted ten-year risk <5% (dark green slice). Without risk modification but increased age by 5 years (No Change scenario), 35% would exceed 5% risk limit (light green and red slices) and 5% would exceed 7.5% threshold (red slice). In comparison, under Reduced Risk Profile, 9% will exceed 5% threshold (light green slice); moreover, at baseline, 13% of the cohort have predicted ten-year risk 5–7.5% (light green). Under Reduced Risk Profile, 30% would exceed 7.5% risk boundary (red slice); while under No Change scenario, 87% would exceed 7.5% risk limit in 5 years (red slice). **B**. The 29% non-diabetic cohort with baseline risk <5% (shown in **A** 1^st^ pie dark green slice) is further divided into three groups with baseline risk <3%, between 3–3.5% and 3.5–5%. For the non-diabetic cohort that has baseline risk between 3–3.5% (orange slice, 1^st^ pie), 2% (light green slice, 2^nd^ column top pie) vs 57% (light green slice, 3^rd^ column top pie) would exceed 5% limit under reduced risk profile scenario vs no change scenario; none exceed the 7.5% boundary. For the cohort that has baseline risk between 3.5–5% (gray slice, 1^st^ pie), 29% (light green slice, 2^nd^ column bottom pie) would exceed the 5% limit under reduced risk profile scenario. Under no change scenario, 94% (light green and red slices, 3^rd^ column bottom pie), would exceed the 5% limit and 18% would even exceed the 7.5% boundary.

Our detailed analysis dataset of NHANES data is attached (Supplement). Table 1 summarizes the baseline characteristics of the NHANES participants. We found that among the non-diabetic cohort, 29% (Dark green slice in Figure 1A 1^st^ pie) had baseline risk of <5%. Amongst these subjects, under No Change scenario, 35% (light green and red slices in Figure 1A 3^rd^ column top pie) would reach or exceed the 5% risk boundary and 5% reach or exceed the 7.5% risk boundary in five years. Under Reduced Risk Profile scenario, 9% (light green slice in Figure 1A 2^nd^ column top pie) would reach or exceed the 5% risk boundary (P<0.0001 vs. no change scenario, Table 2) but none exceeded the 7.5% risk boundary. We also found that 13% of the non-diabetic cohort had baseline risk between 5–7.5% (light green slice in Figure 1A 1^st^ pie). Amongst these subjects, under No Change scenario, 87% would reach or exceed 7.5% and the rest 13% remained between 5–7.5% in 5 years (Figure 1A 3^rd^ column bottom pie). While under Reduced Risk Profile scenario, 30% reached or exceed 7.5% risk despite reasonable risk modification (P<0.0001 vs. no change scenario, Table 2), 50% remained between 5–7.5% and 20% actually had their risk reduced to <5% (Figure 1A 2^rd^ column bottom pie). When we incorporated Hispanics and calculated the risk based on white cohort equations, we find the results remained consistent (Table S2).

**Table 1:** **Baseline characteristics of the NHANES data.**

Variable	Values
# of Participant	1805
Age, yrs	60±10
Total Cholesterol, mg/dl	199±41
HDL Cholesterol, mg/dl	54±17
Blood Pressure, mmHg	133±20
female, %	54
African American, %	38
Caucasian, %	62
Diabetes, %	28
Smoker, %	17
HTN, %	90

Values are n, % or mean ± standard deviation.

**Table 2:** **Compare ten-year risk in five years with and without change in modifiable risk factors.**

	Baseline Ten-Year Risk<5%					Baseline Ten-Year Risk 5–7.5%				
	% of total, n	10-yr Risk ≥5% in 5 yrs		10-yr Risk ≥7.5% in 5 yrs		% of total, n	10-yr Risk ≥5% in 5 yrs		10-yr Risk ≥7.5% in 5 yrs	
		Reduced Risk Profile (% of Baseline Ten-Year Risk<5%, n)	No Change (% of Baseline Ten-Year Risk<5%, n)	Reduced Risk Profile (% of Baseline Ten-Year Risk<5%, n)	No Change (% of Baseline Ten-Year Risk<5%, n)		Reduced Risk Profile (% of Baseline Ten-Year Risk 5–7.5%, n)	No Change (% of Baseline Ten-Year Risk 5–7.5%, n)	Reduced Risk Profile (% of Baseline Ten-Year Risk 5–7.5%, n)	No Change (% of Baseline Ten-Year Risk 5–7.5%, n)
All(n = 1805)	22.33, 403	8.93, 36	36.97, 149***	0.00, 0	5.96, 24***	10.64, 192	81.77, 157	100.00, 192***	30.21, 58	88.54, 170***
Non-DM(n = 1292)	28.79, 372	8.60, 32	35.48, 132***	0.00, 0	5.11, 19***	13.00, 168	79.76, 134	100.00, 168***	29.76, 50	86.90, 146***
AA (n = 426)	23.47, 100	0.00, 0	34.00, 34***	0.00, 0	1.00, 1	12.68, 54	77.78, 42	100.00, 54***	5.56, 3	72.22, 39***
AA Male(n = 196)	4.08, 8	0.00, 0	50.00, 4	0.00, 0	0.00, 0	10.20, 20	95.00, 19	100.00, 20	0.00, 0	60.00, 12***
AA Female(n = 230)	40.00, 92	0.00, 0	32.61, 30***	0.00, 0	1.09, 1	14.78, 34	67.65, 23	100.00, 34***	8.82, 3	79.41, 27***
White(n = 866)	31.41, 272	11.76, 32	36.03, 98***	0.00, 0	6.62, 18***	13.16, 114	80.70, 92	100.00, 114***	41.23, 47	93.86, 107***
White Male(n = 404)	22.77, 92	8.70, 8	46.74, 43***	0.00, 0	5.43, 5	12.13, 49	83.67, 41	100.00, 49**	18.37, 9	97.96, 48***
White Female(n = 462)	38.96, 180	13.33, 24	30.56, 55***	0.00, 0	7.22, 13***	14.07, 65	78.46, 51	100.00, 65***	58.46, 38	90.77, 59***
DM(n = 513)	6.04, 31	12.90, 4	54.84, 17**	0.00, 0	16.13, 5	4.68, 24	95.83, 23	100.00, 24	33.33, 8	100.00, 24***
AA(n = 255)	3.53, 9	0.00, 0	44.44, 4	0.00, 0	22.22, 2	2.35, 6	83.33, 5	100.00, 6	0.00, 0	100.00, 6**
AA Male(n = 107)	0.00, 0	0.00, 0	0.00, 0	0.00, 0	0.00, 0	0.00, 0	0.00, 0	0.00, 0	0.00, 0	0.00, 0
AA Female(n = 148)	6.08, 9	0.00, 0	44.44, 4	0.00, 0	22.22, 2	4.05, 6	83.33, 5	100.00, 6	0.00, 0	100.00, 6**
White(n = 258)	8.53, 22	18.18, 4	59.09, 13*	0.00, 0	13.64, 3	6.98, 18	100.00, 18	100.00, 18	44.44, 8	100.00, 18***
White Male(n = 130)	5.38, 7	14.29, 1	85.71, 6*	0.00, 0	14.29, 1	5.38, 7	100.00, 7	100.00, 7	42.86, 3	100.00, 7
White Female(n = 128)	11.72, 15	20.00, 3	46.67, 7	0.00, 0	13.33, 2	8.59, 11	100.00, 11	100.00, 11	45.45, 5	100.00, 11*

Values are % or n. Reduced Risk Profile, a 10% reduction in total-c and systolic BP, a 10% increase in HDL-c, and no smoking (for those who were smokers); No Change, no change in the other parameters except age increase by five years; Comparisons between Reduced Risk Profile vs No Change were performed using Fisher's Exact Test.

* for P<0.05,

** for P<0.01, and *** for P<0.001.

**Figure 2:**
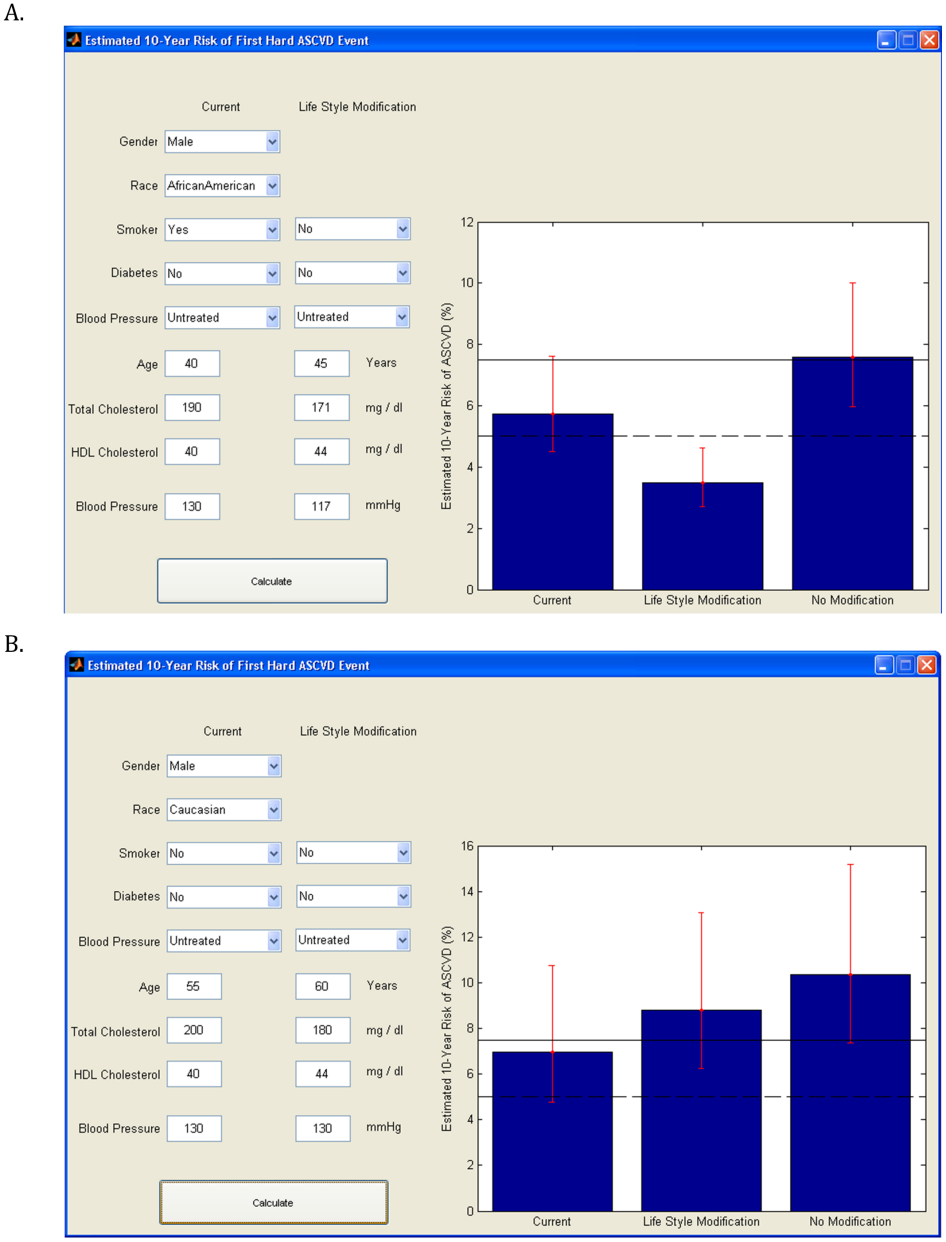
Examples of using the new risk calculator in two cases. Case A indicates that if the patient quits smoking, and improves other risk factors by 10%, the calculated risk at age 45 years is below 5%. In contrast if the baseline risk variables remain the same, and age increases by 5 years, the absolute ten- year risk would be ≥ 7.5% Case B indicates that even if there is 10% improvement in total and HDL-c but no change in BP, the risk will exceed 7.5% regardless of the presumed risk modification.

The 29% non-diabetic cohort with baseline risk <5% (shown as the dark green slice in Figure 1A 1^st^ pie) was further divided into three groups with baseline risk<3% (58%), between 3–3.5% (14%) and 3.5%–5% (28%), as shown in Figure 1B 1^st^ pie. Amongst the non-diabetic subjects with baseline risk between 3–3.5%, under Reduced Risk Profile scenario, 2% would exceed the 5% limit in five years (Figure 2B 2^nd^ column, top pie) while, under No Change scenario, 57% would exceed the 5% risk boundary(Figure 2B, 3^rd^ column, top pie). However, none of these subjects would exceed the 7.5% limit in five years under both scenarios. Furthermore, for non-diabetic subjects with baseline risk between 3–3.5% (gray slice in Figure 1B 1^st^ pie), 29% would exceed the 5% limit but all within the 7.5% limit in five years. With no change risk profile, 94% would exceed the 5% limit (light green and red slices in Figure 1B 3^rd^ column bottom pie) and 18% would even exceed the 7.5% limit.

Moreover, we find that only 6% and 5% of the diabetic cohort had baseline risk of <5% and between 5–7.5%, respectively. Amongst the diabetic participants who were at <5% risk at baseline, under No Change scenario, 16% reached or exceeded 7.5% risk in five years (Table 2). Amongst the same cohort between 5–7.5% baseline risks, under No Change scenario, all reached or exceed 7.5%. While under Reduced Risk Profile scenario, 33% reached or exceed 7.5% risk (Table 2). When we incorporated Hispanics and calculated the risk based on white cohort equations, we find the results remained consistent (Table S2).

### Modified ten- year risk calculator

We provide two examples of realistic case scenarios to demonstrate the versatility of our version of the calculator for personalized medicine:

#### Example 1

African American male, 40 years of age, smoker, non diabetic, total-c 190 mg/dl, HDL-c 40 mg/dl and systolic BP 130 mmHg with no treatment - his baseline ten-year risk is between 5–7.5%. However if he quits smoking, and improves other risk factors by 10%, the calculated risk at age 45 years is below 5%. In contrast if the baseline risk variables remain the same, and age increases by 5 years, the absolute ten-year risk would be ≥ 7.5% (Figure 2A)

#### Example 2

White male, 55 years of age, non smoker, non diabetic, total-c 200 mg/dl, HDL-c 40 mg/dl and systolic BP 130 mmHg with no treatment- his baseline ten-year risk is between 5–7.5%. If there is 10% improvement in total and HDL-c but no change in BP, the risk will exceed 7.5% regardless of the presumed risk modification (Figure 2B).

In Example 1, the individual may decide to be more aggressive with lifestyle modification including smoking cessation, which will preclude statin therapy based on the guidelines. In Example 2, despite reasonable lifestyle modification, the individual likely would exceed the 7.5% threshold for initiating the statin therapy. This patient may therefore be inclined to both life style modification and statin therapy.

## Discussion

Our analysis of the new-pooled cohort equations for ten-year ASCVD risk quantification demonstrates that a substantial number of individuals with borderline risk (either less than 5% or between 5%–7.5% ten-year risk) who are below the treatment thresholds would exceed these thresholds despite presumed reasonable improvements in the modifiable risk factors within the recommended window for reassessing the ten-year risk. These findings may therefore influence the discussion between an individual and the physician regarding initiation of the statin therapy and may also provide an impetus for more aggressive life style modification. Our alternative tool based on the new pooled cohort equations for ten-year risk calculation allows for simultaneously calculating the predicted ten-year risk at certain duration from baseline measurement. This calculation relies on the doctor-patient interactions and incorporates intelligent estimate of the extent of risk factors modification that a patient may achieve with healthy life style with time.

The guideline panel recommends recalculation of estimated ten-year ASCVD risk every four to six years in individuals aged 40–75 years without clinical ASCVD or diabetes suggesting that there may be dynamic changes in the risk profile. Using our tool, one can make a reasonable prediction of the risk profile based on expected trajectory of some of the modifiable risk factors. However the intent of the modified calculator is not to quantify the effects of lifestyle changes on ten-year risk per say. Healthy lifestyle will reduce cardiovascular risk regardless of the calculated risk. The suggested re-evaluation of ten-year risk at four to six year interval is to recalibrate therapies based on clinical evolution.

Here we calculated predicted risk taking into account clinically relevant improvements in the risk profile in five years. As expected, we find that there are a substantial number of people who will remain below the threshold limits for statin therapy after reasonable improvement in modifiable risk factors. However, we also find that there is a significant subset of individuals who will exceed the 5% or the 7.5% thresholds with time regardless of improvement in modifiable risk factors. Onset of atherosclerosis occurs at relatively young age and progresses with time at a variable rate. It is unclear if these individuals who are below the treatment thresholds would benefit with initiation of statin therapy even at baseline. One approach that the panel took in the recent guidelines was to calculate the number needed to treat for benefit and compared it to the number needed to harm due to statin use[1]. They found that for moderate statin therapy, the number needed to treat with statin for benefit is 57 to 67 compared to number needed to harm which is 100 for primary prevention in individuals with ten-year risk between 5% to 7.4%. At ten-year risk corresponding to 3.2%, there appears to be clinical equipoise with no net benefit of moderate intensity statin therapy. Based on this observation and our analysis, statin therapy may be considered for the individuals who are between 3.5%–5% ten-year threshold at baseline but are expected to exceed the 5% or 7.5% threshold with time. For individuals who are between 5–7.5% ten-year risks and are expected to exceed 7.5% risk with time, this may provide a greater acceptability of initiating the statin therapy after discussion with their physicians. An alternative approach in these individuals with borderlines risks may be to test for other risk factors such as calcium score that may help with the decision making. Regardless, either of these approaches needs to be tested in a prospective fashion.

The intent of this manuscript is to promote a more comprehensive discussion amongst the patient and the physician that takes into account the natural history of atherosclerosis, patient preferences and realistic assessment of achievable healthy life style goals. Here we provide an outline for individuals with borderline risk who may be below the threshold limits but may be more inclined to statin therapy and more aggressive life style modification after discussion with their physician.

## Supporting Information

Table S1 Baseline characteristics of the NHANES data.
(DOCX)

Table S2 Compare ten-year risk in five years with and without change in modifiable risk factors (with Hispanic).
(DOCX)

Calculator S1 Modified ten-year risk calculator.
(EXE)

Calculator Instruction S1 Instruction for the modified ten-year risk calculator.
(DOCX)

Data S1 NHANES dataset 2005–2010 without Hispanics.
(XLSX)

Data S2 NHANES dataset 2005–2010 with Hispanics.
(XLSX)
