# Supplementary material for: Modified Treatment Approach Using Cardiovascular Disease Risk Calculator for Primary Prevention
Source: PLoS One. 2014 Aug 13;9(8):e104478. doi: 10.1371/journal.pone.0104478 (PMC4131882; doi:10.1371/journal.pone.0104478)
Supplement: Table S1 — Baseline characteristics of the NHANES data. (DOCX) [file pone.0104478.s001.docx]

| **Table S1: Baseline characteristics of the NHANES data** | |
| --- | --- |
| Variable | Values |
| # of Participant | 2355 |
| Age, yrs | 60±10 |
| Total Cholesterol, mg/dl | 200±41 |
| HDL Cholesterol, mg/dl | 53±17 |
| Blood Pressure, mmHg | 133±20 |
| female, % | 55 |
| African American, % | 29 |
| Caucasian, % | 71 |
| Diabetes, % | 32 |
| Smoker, % | 16 |
| HTN, % | 89 |
| **Values are n, % or mean ± standard deviation.** | |
