# Supplementary material for: Modified Treatment Approach Using Cardiovascular Disease Risk Calculator for Primary Prevention
Source: PLoS One. 2014 Aug 13;9(8):e104478. doi: 10.1371/journal.pone.0104478 (PMC4131882; doi:10.1371/journal.pone.0104478)
Supplement: Table S2 — Compare ten-year risk in five years with and without change in modifiable risk factors (with Hispanic). (DOCX) [file pone.0104478.s002.docx]

| Table S2: Compare ten-year risk in five years with and without change in modifiable risk factors (with Hispanic) | | | | | | | | | | | |
| --- | --- | --- | --- | --- | --- | --- | --- | --- | --- | --- | --- |
|  | Baseline Ten-Year Risk<5% | | | | |  | Baseline Ten-Year Risk 5-7.5% | | | | |
|  | % of total, n | 10-yr Risk ≥5% in 5yrs | | 10-yr Risk ≥7.5% in 5yrs | |  | % of total, n | 10-yr Risk ≥5% in 5yrs | | 10-yr Risk ≥7.5% in 5yrs | |
|  |  | Reduced Risk Profile (% of Baseline Ten-Year Risk<5%, n) | No Change (% of Baseline Ten-Year Risk<5%, n) | Reduced Risk Profile (% of Baseline Ten-Year Risk<5%, n) | No Change (% of Baseline Ten-Year Risk<5%, n) |  |  | Reduced Risk Profile (% of Baseline Ten-Year Risk 5-7.5%, n) | No Change (% of Baseline Ten-Year Risk 5-7.5%, n) | Reduced Risk Profile (% of Baseline Ten-Year Risk 5-7.5%, n) | No Change (% of Baseline Ten-Year Risk 5-7.5%, n) |
| All(n=2355) | 22.76, 536 | 10.45, 56 | 37.69, 202*** | 22.76, 536 | 10.45, 56*** |  | 10.57, 249 | 82.33, 205 | 100.00, 249*** | 30.92, 77 | 88.76, 221*** |
| Non-DM(n=1601) | 30.17, 483 | 10.35, 50 | 35.40, 171*** | 30.17, 483 | 10.35, 50*** |  | 13.05, 209 | 80.86, 169 | 100.00, 209*** | 30.14, 63 | 88.04, 184*** |
| AA (n=426) | 23.47, 100 | 0.00, 0 | 34.00, 34*** | 23.47, 100 | 0.00, 0 |  | 12.68, 54 | 77.78, 42 | 100.00, 54*** | 5.56, 3 | 72.22, 39*** |
| AA Male(n=196) | 4.08, 8 | 0.00, 0 | 50.00, 4 | 4.08, 8 | 0.00, 0 |  | 10.20, 20 | 95.00, 19 | 100.00, 20 | 0.00, 0 | 60.00, 12*** |
| AA Female(n=230) | 40.00, 92 | 0.00, 0 | 32.61, 30*** | 40.00, 92 | 0.00, 0 |  | 14.78, 34 | 67.65, 23 | 100.00, 34*** | 8.82, 3 | 79.41, 27*** |
| White(n=1175) | 32.60, 383 | 13.05, 50 | 35.77, 137*** | 32.60, 383 | 13.05, 50*** |  | 13.19, 155 | 81.94, 127 | 100.00, 155*** | 38.71, 60 | 93.55, 145*** |
| White Male(n=532) | 22.18, 118 | 9.32, 11 | 44.92, 53*** | 22.18, 118 | 9.32, 11* |  | 11.28, 60 | 85.00, 51 | 100.00, 60** | 16.67, 10 | 98.33, 59*** |
| White Female(n=643) | 41.21, 265 | 14.72, 39 | 31.70, 84*** | 41.21, 265 | 14.72, 39*** |  | 14.77, 95 | 80.00, 76 | 100.00, 95*** | 52.63, 50 | 90.53, 86*** |
| DM(n=754) | 7.03, 53 | 11.32, 6 | 58.49, 31*** | 7.03, 53 | 11.32, 6* |  | 5.31, 40 | 90.00, 36 | 100.00, 40 | 35.00, 14 | 92.50, 37*** |
| AA(n=255) | 3.53, 9 | 0.00, 0 | 44.44, 4 | 3.53, 9 | 0.00, 0 |  | 2.35, 6 | 83.33, 5 | 100.00, 6 | 0.00, 0 | 100.00, 6** |
| AA Male(n=107) | 0.00, 0 | 0.00, 0 | 0.00, 0 | 0.00, 0 | 0.00, 0 |  | 0.00, 0 | 0.00, 0 | 0.00, 0 | 0.00, 0 | 0.00, 0 |
| AA Female(n=148 ) | 6.08, 9 | 0.00, 0 | 44.44, 4 | 6.08, 9 | 0.00, 0 |  | 4.05, 6 | 83.33, 5 | 100.00, 6 | 0.00, 0 | 100.00, 6** |
| White(n=499) | 8.82, 44 | 13.64, 6 | 61.36, 27*** | 8.82, 44 | 13.64, 6 |  | 6.81, 34 | 91.18, 31 | 100.00, 34 | 41.18, 14 | 91.18, 31*** |
| White Male(n=220) | 4.09, 9 | 11.11, 1 | 88.89, 8** | 4.09, 9 | 11.11, 1 |  | 4.55, 10 | 100.00, 10 | 100.00, 10 | 40.00, 4 | 100.00, 10* |
| White Female(n=279) | 12.54, 35 | 14.29, 5 | 54.29, 19*** | 12.54, 35 | 14.29, 5 |  | 8.60, 24 | 87.50, 21 | 100.00, 24 | 41.67, 10 | 87.50, 21** |
| Values are % or n. Reduced Risk Profile, a 10% reduction in total-c and systolic BP, a 10% increase in HDL-c, and no smoking (for those who were smokers); No Change, no change in the other parameters except age increase by five years; Comparisons between Reduced Risk Profile vs No Change were performed using Fisher’s Exact Test. * for P < 0.05, ** for P < 0.01, and *** for P < 0.001. | | | | | | | | | | | |
