# Supplementary material for: Modified Treatment Approach Using Cardiovascular Disease Risk Calculator for Primary Prevention
Source: PLoS One. 2014 Aug 13;9(8):e104478. doi: 10.1371/journal.pone.0104478 (PMC4131882; doi:10.1371/journal.pone.0104478)
Supplement: Calculator Instruction S1 — Instruction for the modified ten-year risk calculator. (DOCX) [file pone.0104478.s004.docx]

**INSTRUCTIONS FOR THE RISK CALCULATOR VER1**

The application is written in Matlab and compiled in Microsoft compiler. This application is compatible with Microsoft windows operating system.

Open the zipped folder and run the program. Default location is *C:\ProgramFiles\ten_year_risk_gui_tool_paper*.

Check the box for short cut to desktop if so desire.

Once installed the program appears in task bar menu under list of programs listed as ten_year_risk_gui tool_paper (or on the desktop).

When you open the application: default window is as below


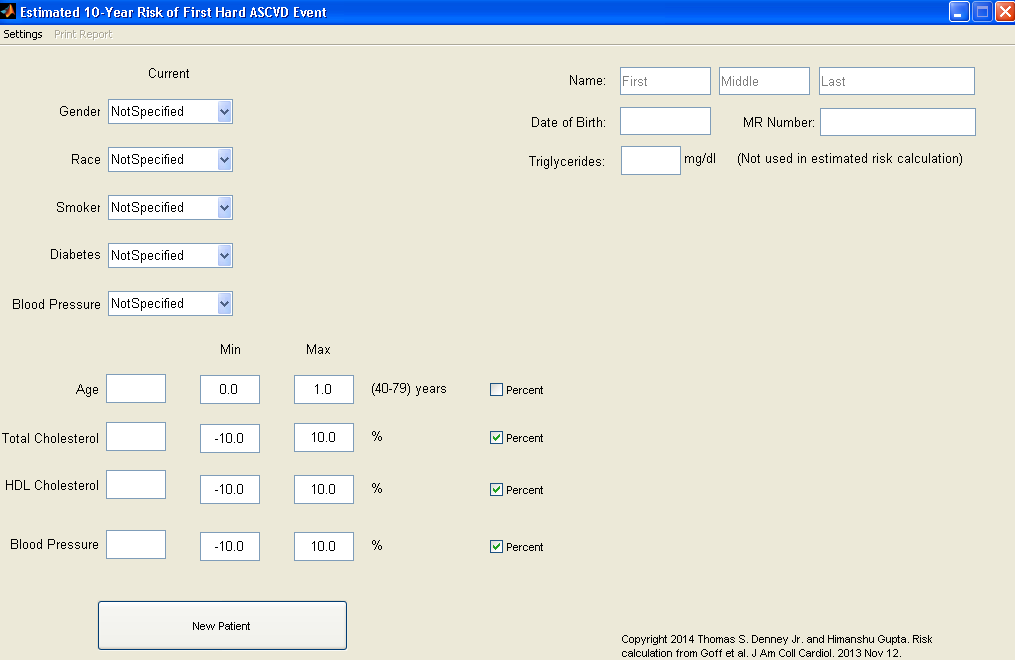


Patient demographics can be inserted that is useful to generate a report.

Input values in boxes with white color.

For ten-year risk calculation, LDL-cholesterol should be 70-189 mg/dl.

Input limits for continuous parameters are as follows:

Age: 40-79 years

Total Cholesterol: 130 – 320 mg/dl

HDL-cholesterol: 20 – 100 mg/dl

Systolic Blood Pressure: 90 - 200 mmHg

If the input values for these continuous parameters are outside the defined limits, the lowest or the highest values corresponding to that variable is input as a default.

The **min** and **max** denotes that uncertainty of the measurement, which can be customized to a particular clinical situation.

The default values are 0-1 years for age, ± 10% for total-cholesterol, HDL-cholesterol and systolic blood pressure.

**Note**: For age, the permissible uncertainty is 0-1 years. It can also be input in percent.

After inputting all the data, press ‘Enter’ or ‘Tab’ key.. It will immediately display the ten year risk and the upper and lower limits based on uncertainty of measurements.


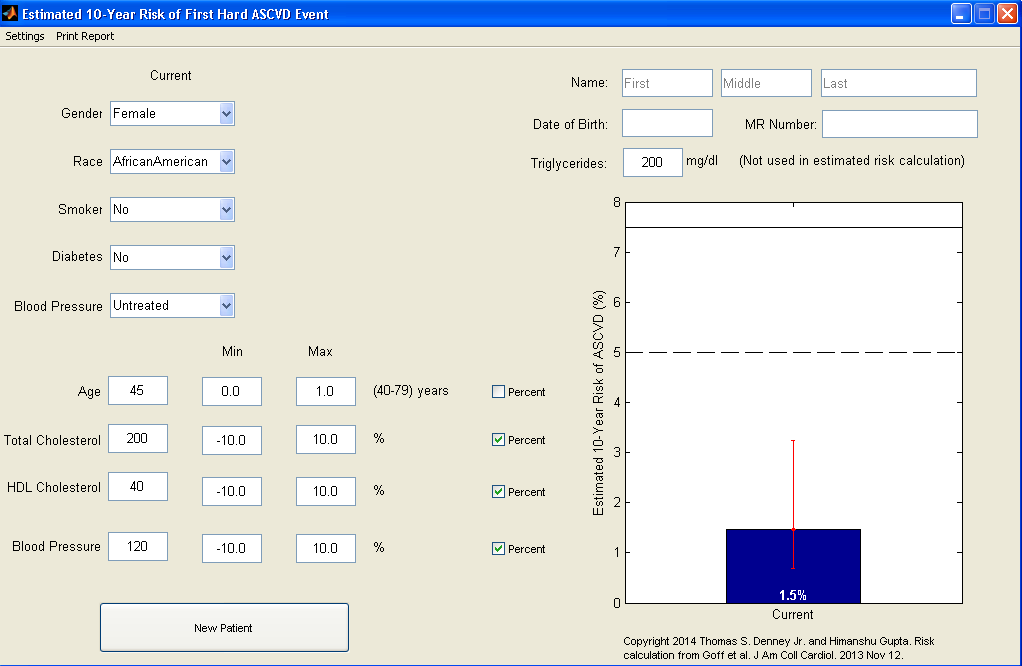


To edit the values, just enter the correct values in their respective boxes.

To set prediction parameters, go to **Settings**

Select: Set prediction parameters.


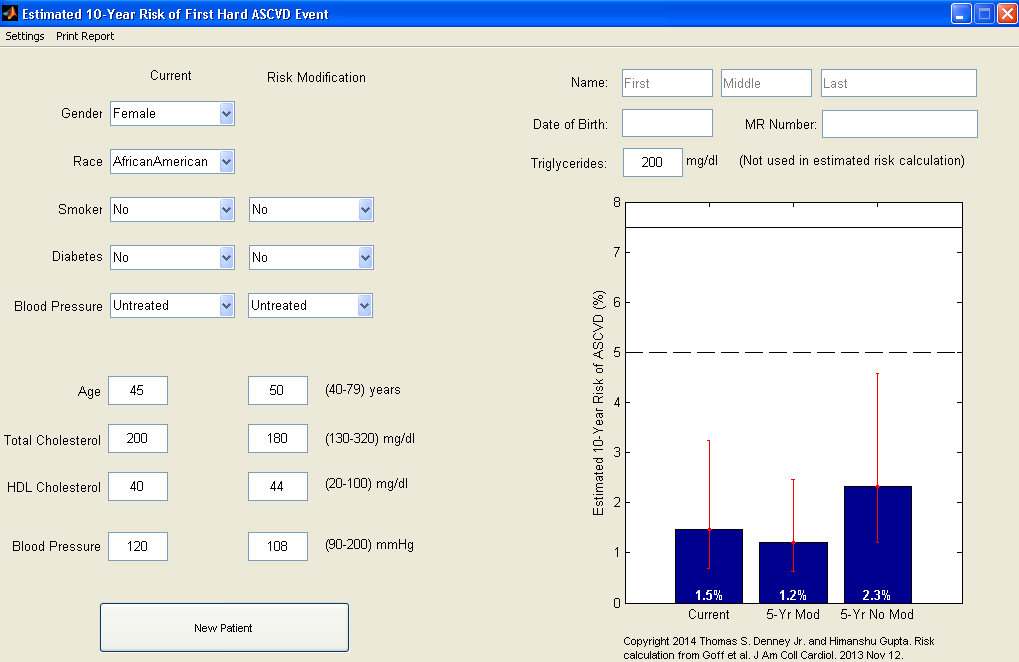


As a default, predicted ten –year risk five years from baseline measurements is depicted under two scenarios.

**Current**: refers to baseline ten-year risk

**5-Yr Mod**: refers to predicted ten-year risk five years from baseline with 10% reduction in total-cholesterol and systolic blood pressure and 10% increase in HDL-cholesterol. Also if smoker, it changes to non smoker.

**5-Yr No Mod**: refers to predicted ten-year risk five years from baseline with no change in parameters except increase in age by five years.

**Note**: for calculating modified risk, the values can be input based on reasonable estimate of the changes in various parameters.

A report can be generated. Go to print report which allows for saving the report in *.txt* format.

For a new patient, click on the ‘New Patient’ tab. It will ask to confirm and once confirmed, previous data will be wiped out and the new data can be input.
